# Adverse Events as a Function of Biological Sex in a Multicenter Clinical Trial of Melanoma Vaccines

**DOI:** 10.3390/cancers16223882

**Published:** 2024-11-20

**Authors:** Catherine E. Lyons, Ruyun Jin, Aaron D. Smith, Hong Zhu, Craig L. Slingluff

**Affiliations:** 1 Department of Surgery, University of Virginia School of Medicine, Charlottesville, VA 22903, USA; 2 Department of Public Health Sciences, Division of Biostatistics, University of Virginia School of Medicine, Charlottesville, VA 22903, USA; 3 Department of Surgery, Division of Surgical Oncology, University of Virginia, Charlottesville, VA 22903, USA; cls8h@uvahealth.org

**Keywords:** metastatic melanoma, clinical trial, peptide vaccine, cancer, adverse events, treatment-related adverse events, biological sex

## Abstract

**Background/Objective:** Biological females experience more autoimmune disease than males and more treatment-related adverse events (TRAEs) after immune checkpoint blockade therapy. However, little is known about sex-related differences in TRAEs after cancer vaccines. **Methods**: The Mel44 clinical trial (NCT00118274) enrolled 167 eligible patients with high-risk melanoma to treatment with either of two melanoma multipeptide vaccines. We hypothesized that females would experience higher rates and grades of TRAEs. TRAE rates and grades were compared between sexes, with adjustment for multiple comparisons, and with mixed-effects models. **Results**: Multiple sex-related differences in TRAE rate and grade were observed in unadjusted comparisons, but only hyperglycemia and hypopigmentation were significantly higher-grade by sex after correcting for multiple comparisons: they were increased in males. In mixed-effect models, vaccination strategy, but not patient sex, was independently associated with TRAE rates and grades. **Conclusions**: These data do not support our hypothesis that TRAEs would be increased in females. Vaccine safety was supported for both males and females.

## Simple Summary

The impacts of biological sex on cancer treatment outcomes are understudied, but differences in immune-related adverse events and oncologic outcomes have been associated with biological sex for patients with melanoma receiving checkpoint blockade therapy. In a recent trial, we also identified a difference in clinical outcomes between biological female and male patients with metastatic melanoma who received an experimental melanoma vaccine. Here, we examine whether treatment-related adverse events with the vaccine differ by biological sex.

## 1. Introduction

Recent data highlight the promise of cancer vaccines for melanoma, and likely for other cancers [1,2,3]. Treatment-related adverse events (TRAEs) attributed to vaccines against human cancers are commonly limited to grade-1 and -2 severity but can occasionally be more serious and dose-limiting [1]. Therefore, although these TRAEs are not generally dose-limiting in the context of vaccines against human cancer, they are important to study because they may reflect autoimmune disease or predict potential clinical benefit [4,5,6,7,8].

The impact of biological sex on clinical outcomes is a high priority for the National Institutes of Health but is understudied. We recently identified sex-related differences in clinical benefit, as biological males have better durable long-term survival than females receiving a multipeptide melanoma vaccine [3]. A similar trend favoring males was identified in patients receiving checkpoint blockade cancer therapy for metastatic melanoma and other advanced cancers [9,10]. Further, other cancer therapies including immunotherapy have induced more frequent general TRAEs and immune-related TRAEs in females than in males [11,12]. Very little is known about the sex-related differences in TRAEs induced by cancer vaccines. Detailed analysis of differences by sex in incidence and grade of TRAEs in cancer vaccine therapy might help to guide the risk–benefit ratio discussion.

The objective of this current study was to determine whether there are differences in TRAEs as a function of biological sex in the context of multipeptide melanoma vaccines, independent of age, American Joint Committee on Cancer (AJCC) stage, and treatment arm. We examined the sex-related differences in TRAEs in the context of the Mel44 peptide vaccine trial (NCT00118274), in which there was a difference in clinical benefit between males and females [3]. We hypothesized that biological females would experience higher rates and grades of TRAEs.

## 2. Materials and Methods

### 2.1. Clinical Trial Design

Mel44 was a multicenter randomized trial approved by the institutional review board designed to test the safety and immunogenicity of two different peptide vaccine combinations as well as the potential for clinical benefit with the addition of low-dose cyclophosphamide (CY) pretreatment, for which the trial design and primary results have been reported [10]. Briefly, for the study, we enrolled patients with resected high-risk melanoma, AJCC (v6) stage IIB-IV, who were clinically free of disease and randomized these patients equally among four treatment arms in a 2 × 2 design to either of two peptide vaccine regimens, with or without low-dose CY pretreatment. Both vaccine regimens contained 12 class 1 major histocompatibility complex (MHC)-restricted melanoma peptides stimulating CD8+ T cells (12MP), but the vaccine regimens differed in the peptides designed to stimulate CD4+ T cells. Patients on arms A and B received a nonspecific tetanus helper peptide (Tet); patients on arms C and D received six melanoma-associated class 2 MHC-restricted melanoma helper peptides to stimulate CD4+ T cells (6 MHP) (Supplemental Table S1) [13].

### 2.2. TRAE Data Collection

The trial was monitored continuously for TRAEs with National Cancer Institute Common Terminology Criteria for Adverse Events (CTCAE) version 3.0 (v3). TRAEs were reviewed weekly by patient interview with a study clinician and recorded in the case report forms and clinical record. TRAEs were given one of the following labels according to the study clinician’s assessment of relatedness to vaccine treatment: “unrelated”, “unlikely”, “possible”, “probable”, or “definite”, according to National Cancer Institute Guidelines for Investigators [14]. Attributions of “possible”, “probable”, or “definite” were deemed treatment-related. Protocol treatment was to be discontinued for unexpected grade 3, ocular grade 1, allergic grade 2, or higher TRAEs. TRAE data have been stored in the University of Virginia C3TO Cancer Center clinical trials office database.

### 2.3. Data Analyses

The number of TRAEs, organized by CTCAE v3 toxicity category/unique descriptions, was reported in the study population. The maximum grade of any TRAE for each patient, the number of TRAEs for each patient, and the maximum grade of unique TRAEs for each patient were extracted. Patient data were organized by the maximum grade of any TRAEs for that patient, by study arm, and by biological sex. Only TRAEs that occurred in more than 5% of the population were further analyzed. The proportion of patients with each grade of TRAE (maximum per patient), as well as the cumulative proportions of patients with one or more different TRAEs, was plotted on cumulative frequency curves for each sex. The incidence rates for each of the unique TRAEs were compared between the biological sexes using chi-square test or Fisher’s exact test as appropriate. The total number of TRAEs and the average grade for each TRAE were compared between males and females by a *t*-test or Wilcoxon rank sum test, as appropriate. *p*-values were adjusted for multiple comparisons using the Benjamani–Hochberg procedure to control the false discovery rate (FDR). Using a linear mixed-effects model with patient and type of TRAE as the random effects, the incidence rate of TRAEs was modeled as a function of biological sex, age, AJCC stage, and treatment arm. Maximum TRAE grade was modeled as a function of biological sex, age, AJCC staging class, and treatments using a mixed-effect ordinal logistic regression with patient and type of TRAE as random effects. (*p* < 0.05) was considered statistically significant. Statistical analyses were performed using R 4.2.3 software (R Foundation for Statistical Computing, Vienna, Austria) with packages lme4, car, and ordinal.

## 3. Results

One hundred seventy patients were enrolled in the Mel44 clinical trial. Adverse events were evaluated for the entire cohort, including three patients found ineligible on post-review [13]. The treatment arms had similar numbers of patients (41, 43, 42, and 44 for arms A–D, respectively), with males predominating (67%). 

**Figure 1:**
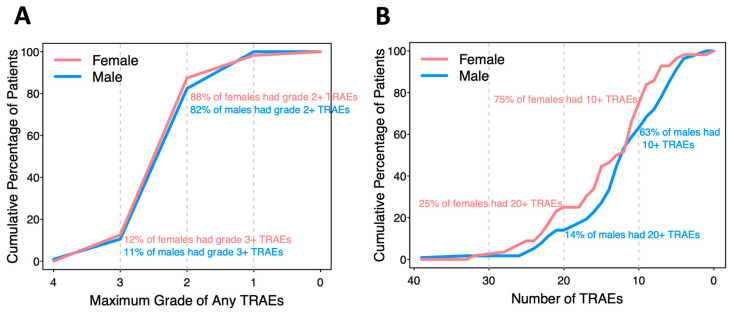
Incidence and grades of TRAEs on Mel44 trial in aggregate, as a function of biological sex: (**A**) cumulative incidence of 42 most frequent TRAEs by sex; (**B**) cumulative maximum grade of 42 most frequent TRAEs by sex.

There were 2648 reported TRAEs involving almost every patient (99.4%), with 126 unique CTCAE descriptions ranging from grade 1 to 4. In the analysis of grade, patients who did not experience a TRAE were assigned grade 0 for that TRAE. Some TRAEs were reported multiple times (up to four) in the same patient. No treatment-related deaths or deaths in the study occurred. When a TRAE was reported more than once for a patient, the maximum grade was recorded, and the numbers with maximum grades 1–4 were 1770 (81%), 368 (17%), 39 (1.8%), and 1 (0.05%), respectively. Forty-two TRAEs occurred in at least 5% of participants and are the focus of comparisons by biological sex. The percentages of patients with maximum grade 4, 3, 2, or 1 of any TRAE in each patient are presented in a cumulative incidence curve (Figure 1A). In total, 12% of females and 11% of males had at least one TRAE grade 3 or above, while 88% of females and 82% of males had at least one TRAE grade 2 or above.

Most of the patients experienced multiple TRAEs (Figure 1B). In total, 25% of females and 14% of males experienced 20 or more different TRAEs; 75% of females and 63% of males experienced 10 or more different TRAEs. Each male and female experienced an average of 12 or 14 TRAEs, respectively (*p* = 0.077).

The total TRAEs by grade in the overall study population, as well as by study arms and sex, are presented in Supplemental Table S2. The rates of patients with grade 0–1, grade 2, or grade 3–4 TRAEs were similar by sex in arms A, B, and C. In arm D, females trended toward higher grades of TRAEs than males (*p* = 0.052).

**Figure 2:**
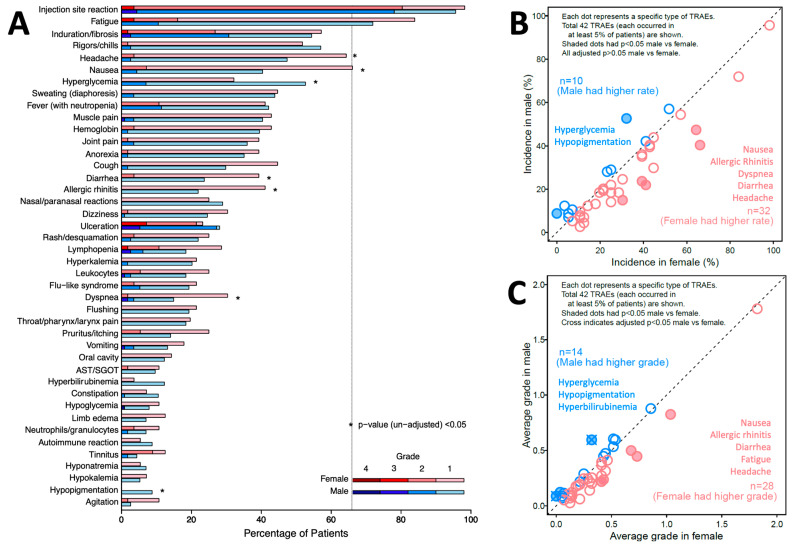
Incidence and maximum grades for each of the 42 most frequent TRAEs in the Mel44 trial: (**A**) incidence of each of the 42 most frequent TRAEs, organized by sex and grade; (**B**) incidence rates for each of the 42 most frequent TRAEs by sex; (**C**) mean of the maximum grades for each of the 42 most frequent TRAEs among males and females.

TRAE incidence is also presented by sex and grade (Figure 2A). The distribution of grade was different between sexes for eight TRAEs. Four TRAEs occurred in more than half of patients, for both females and males: injection site reaction (98%, 96%, respectively), fatigue (84%, 72%), induration or fibrosis (57%, 54%), and rigors/chills (52%, 57%). Thirty-two unique TRAEs occurred more frequently in females, while ten occurred more frequently in males. Before adjustment for multiple comparisons, five of these TRAEs were more frequent (*p* < 0.05) in females: nausea (*p* = 0.002), allergic rhinitis (*p* = 0.009), dyspnea (*p* = 0.018), diarrhea (*p* = 0.035), and headache (*p* = 0.038). Two were more frequent in males: hyperglycemia (*p* = 0.012) and hypopigmentation (*p* = 0.032), as marked with asterisks in Figure 2A. However, with adjustment, *p* > 0.05 for all those TRAEs (Figure 2B).

TRAE severity was calculated as the mean of the maximum grades of each TRAE across the population, assigning grade 0 for those without that TRAE. Females experienced higher average grades in 28 unique TRAEs, while males experienced higher average grades in 14 TRAEs (Figure 2C). Without correcting for other covariates or *p*-value adjustment, females experienced significantly higher average grades for five TRAEs: nausea (*p* = 0.004), allergic rhinitis (*p* = 0.015), diarrhea (*p* = 0.028), fatigue (*p* = 0.046), and headache (*p* = 0.048). Males experienced significantly higher average grades in three TRAEs, including hypopigmentation (*p* = 0.001), hyperglycemia (*p* = 0.002), and hyperbilirubinemia (*p* = 0.030). Adjusted (*p* < 0.05) were observed for increased hypopigmentation (*p* = 0.042) and hyperglycemia (*p* = 0.042) in males (Figure 2C).

Grade 1–2 TRAEs are usually well tolerated and not dose-limiting toxicities (DLTs). Severe TRAEs could impact patient outcomes, and when considered DLTs, they limit the completion of investigational therapy. Among the 170 patients, we previously reported that 15 (8.8%) experienced grade 3–4 treatment-related DLTs. We have now identified that they represented 11 males and 4 females. These represented 9.7% of males and 7.1% of females (*p* = 0.59). Thus, there was no sex-related difference in participants with grade 3–4 DLTs. 

The rates of the 42 TRAEs by sex were assessed in a linear mixed-effects model, adjusting by age, AJCC staging, and vaccine treatments. Considering patient and TRAE type as random effects, biological sex did not influence the incidence of TRAE (*p* = 0.105). However, there was a 7% increase in the odds of the incidence of TRAEs for patients treated with 12MP + Tet compared to patients treated with 12MP + 6MHP (*p* = 0.001, Table 1). Similarly, in an ordinal mixed-effects logistic regression analysis, biological sex also did not influence the grades of TRAE (*p* = 0.358). However, there was a 60% increase in the odds of experiencing higher-grade TRAEs for patients who received 12MP + Tet compared to patients who received 12MP + 6MHP (*p* < 0.001, Table 2).

## 4. Discussion

We identified interesting trends for differences in the incidence and grades of TRAEs as a function of biological sex in this trial. There was a trend to higher numbers of TRAEs in females (*p* = 0.077, Figure 1A) and to higher grades of TRAEs overall (*p* = 0.052, Figure 1B). Initial assessments identified eight (19%) TRAEs with different incidences in females or males, but none were different with adjustment for multiple comparisons (Figure 2B). There were significantly higher grades of hyperglycemia and hypopigmentation in males after correction for multiple comparisons (Figure 2C). Overall, however, mixed-effect models did not support differences in TRAEs by biological sex when controlling for other factors. On the other hand, the modeling supported the notion that patients vaccinated with 12MP + Tet rather than those vaccinated with 12MP + 6MHP experienced greater TRAE frequency (*p* = 0.001) and severity (*p* < 0.001).

In prior work, we reported that selected inflammatory TRAEs were associated with higher rates of immune response across several vaccine trials, and there is a reported association in the literature of inflammatory TRAEs with response to immunotherapy treatments for melanoma [4,15,16,17]. Stronger immune responses may be associated with greater local toxicity due to inflammation at the vaccine sites and systemic toxicities mediated by cytokine release. However, we have recently identified more favorable long-term overall survival in patients in the Mel44 trial who were vaccinated with 12MP + 6 MHP than those vaccinated with 12MP + Tet, specifically in males, despite lower CD8+ T cell response rates with the 12MP + 6MHP vaccines [3,13]. Relationships between TRAEs and clinical outcome are likely complex, as TRAEs may reflect nonspecific inflammatory effects as well as antigen-specific reactivities.

Our findings differ from reported experience with RNA vaccines for COVID-19, where TRAEs were more common in females than males, including fatigue, headache, vaccine site reactions, joint pain, and others, many of which were also higher in younger patients [18]. Similarly, other studies have identified increased TRAEs in females after COVID-19 RNA vaccines [19]. Similarly, women were more than 3-fold more likely to report AEs after influenza vaccines than men, an effect that was associated with biological sex rather than self-reported gender [20]. Also, a large study of national cooperative group clinical trials identified 66% higher rates of severe AE symptoms in patients receiving immunotherapy, and also higher rates of severe hematologic AEs with chemotherapy or immunotherapy [12]. Thus, our original hypothesis for greater AEs in women was consistent with these and other reports. We did identify non-significant trends to increased overall AE rates and severity in women, and increased frequencies of some individual AEs in females or males before correcting for multiple comparisons, but when correcting for multiple comparisons and mixed-effect modeling, differences in immune response by sex were not significant. The peptide vaccines used in this trial differ qualitatively from the RNA vaccines for COVID-19 and from influenza vaccines. These differences may have a role in the lack of significant changes in TRAE rates as a function of biological sex in this study.

This study’s limitations include the analysis of just one clinical trial. However, this clinical trial was multicenter and randomized, and included a sizeable number of patients for a phase II trial. It is possible that in a larger study, some of the TRAEs may differ in frequency even after correcting for multiple comparisons. However, this analysis supports the safety of the multipeptide vaccines in both males and females and does not support the hypothesis that TRAEs were more frequent in females. The power of the analysis to identify subgroup differences in TRAE rates was supported by our finding, in mixed models controlling for sex, age, stage and treatment, that TRAEs were significantly associated with the vaccine regimen, including tetanus toxoid peptide.

## 5. Conclusions

Sex-related differences have been observed in clinical outcome and adverse events experienced with various immune therapies. Thus, for patients in our clinical trial of melanoma peptide vaccines, we hypothesized that there would be increased AEs in females. However, our data do not support this hypothesis. Instead, vaccine safety was supported for both males and females. These findings raise the possibility that cancer vaccines generally, or the vaccine approaches used in this trial in particular, may differ from some other vaccines or other cancer immunotherapies by inducing similar AE rates for both sexes.

## Figures and Tables

**Table 1:** Linear mixed-effects model for the rates of the 42 TRAEs.

Covariate	Detail	*p*-Value	Odds Ratio (95% CI)
Sex	Female vs. male	0.105	1.03 (0.99, 1.08)
Arm (Treatment)	A + B (12MP + Tet) vs. C + D (12MP + 6MHP)	0.001	1.07 (1.03, 1.11)

**Table 2:** Ordinal mixed-effects logistic regression model for the grade of the 42 TRAEs.

Covariate	Detail	*p*-Value	Odds Ratio (95% CI)
Age	by 1 year	0.028	0.99 (0.98, 1.00)
Sex	Female vs. male	0.358	1.15 (0.86, 1.53)
Arm (Treatment)	A + B (12MP + Tet) vs. C + D (12MP + 6MHP)	<0.001	1.60 (1.22, 2.10)

## Data Availability

The raw data supporting the conclusions of this article will be made available by the authors on request.

## Supplementary Materials

The following supporting information can be downloaded at: https://www.mdpi.com/article/10.3390/cancers16223882/s1, Table S1: Treatments assigned for patients enrolled in Mel44 trial in 4 study arms; Table S2: TRAE counts in Mel44 clinical vaccine trial, organized by grade, vaccine arm, and biological sex.
