# Supplementary material for: Adverse Events as a Function of Biological Sex in a Multicenter Clinical Trial of Melanoma Vaccines
Source: Cancers (Basel). 2024 Nov 20;16(22):3882. doi: 10.3390/cancers16223882 (PMC11592796; doi:10.3390/cancers16223882)
Supplement: Supplementary file 1 [file cancers-16-03882-s001.zip › cancers-3294021-supplementary.pdf]

Supplemental Tables

Table S1. Treatments assigned for patients enrolled on Mel44 trial in 4 study arms

Mel 44 Study Arm	Peptides to stimulate CD8 T cells	Peptides to stimulate CD4 T cells	Cyclophosphamide pre-treatment
A	12MP	Tet	No
B	12MP	Tet	Yes
C	12MP	6MHP	No
D	12MP	6MHP	Yes

12MP = 12 class 1 major histocompatibility complex-restricted melanoma peptides stimulating $CD8^+$ T cells;
Tet = nonspecific tetanus helper peptide;
6MHP = six melanoma-associated class 2 major histocompatibility complex-restricted melanoma helper peptides stimulating $CD4^+$ T cells

Table S2. TRAE counts in Mel44 clinical vaccine trial, organized by grade, vaccine arm and biological sex

		Total*	Male	Female	p-value
**Overall**	*Patient Numbers (% of total)*	*170*	*114 (67%)*	*56 (33%)*	
	N without TRAEs	1 (1%)	0 (0%)	1 (2%)	0.421
	N with grade 1 TRAEs	26 (15%)	20 (18%)	6 (11%)	
	N with grade 2 TRAEs	124 (73%)	82 (72%)	42 (75%)	
	N with grade 3 TRAEs	18 (11%)	11 (10%)	7 (12%)	
	N with grade 4 TRAEs	1 (1%)	1 (1%)	0 (0%)	
**Arm A**	*Patient Numbers (% of total)*	*41*	*28 (68%)*	*13 (32%)*	
	N with grade 1 TRAEs or no TRAEs	5 (12%)	5 (18%)	0 (0%)	0.309
	N with grade 2 TRAEs	30 (73%)	19 (68%)	11 (85%)	
	N with grade 3 or 4 TRAEs	6 (15%)	4 (14%)	2 (15%)	
**Arm B**	*Patient Numbers (% of total)*	*43*	*26 (60%)*	*17 (40%)*	
	N with grade 1 TRAEs or no TRAEs	2 (5%)	1 (4%)	1 (6%)	0.730
	N with grade 2 TRAEs	33 (77%)	19 (73%)	14 (82%)	
	N with grade 3 or 4 TRAEs	8 (19%)	6 (23%)	2 (12%)	
**Arm C**	*Patient Numbers (% of total)*	*42*	*29 (69%)*	*13 (31%)*	
	N with grade 1 TRAEs or no TRAEs	12 (29%)	6 (21%)	6 (46%)	0.177
	N with grade 2 TRAEs	28 (67%)	22 (76%)	6 (46%)	
	N with grade 3 or 4 TRAEs	2 (5%)	1 (3%)	1 (8%)	
**Arm D**	*Patient Numbers (% of total)*	*44*	*31 (70%)*	*13 (30%)*	
	N with grade 1 TRAEs or no TRAEs	8 (18%)	8 (26%)	0 (0%)	0.052
	N with grade 2 TRAEs	33 (75%)	22 (71%)	11 (85%)	
	N with grade 3 or 4 TRAEs	3 (7%)	1 (3%)	2 (15%)	

*Total TRAEs were previously reported, but the breakdown by gender was not reported.
